# Morphological and Hemodynamic Analysis of Mirror Posterior Communicating Artery Aneurysms

**DOI:** 10.1371/journal.pone.0055413

**Published:** 2013-01-31

**Authors:** Jinyu Xu, Ying Yu, Xi Wu, Yongfa Wu, Che Jiang, Shengzhang Wang, Qinghai Huang, Jianmin Liu

**Affiliations:** 1 Department of Neurosurgery, Changhai Hospital, Second Military Medical University, Shanghai, China; 2 Department of Mechanics and Engineering Science, Fudan University, Shanghai, China; University of Cambridge, United Kingdom

## Abstract

**Background and Purpose:**

Hemodynamic factors are commonly believed to play an important role in the pathogenesis, progression, and rupture of cerebral aneurysms. In this study, we aimed to identify significant hemodynamic and morphological parameters that discriminate intracranial aneurysm rupture status using 3-dimensional-angiography and computational fluid dynamics technology.

**Materials and Methods:**

3D-DSA was performed in 8 patients with mirror posterior communicating artery aneurysms (Pcom-MANs). Each pair was divided into ruptured and unruptured groups. Five morphological and three hemodynamic parameters were evaluated for significance with respect to rupture.

**Results:**

The normalized mean wall shear stress (WSS) of the aneurysm sac in the ruptured group was significantly lower than that in the unruptured group (0.52±0.20 versus 0.81±0.21, P = .012). The percentage of the low WSS area in the ruptured group was higher than that in the unruptured group (4.11±4.66% versus 0.02±0.06%, P = .018). The AR was 1.04±0.21 in the ruptured group, which was significantly higher than 0.70±0.17 in the unruptured group (P = .012). By contrast, parameters that had no significant differences between the two groups were OSI (P = .674), aneurysm size (P = .327), size ratio (P = .779), vessel angle (P = 1.000) and aneurysm inclination angle (P = 1.000).

**Conclusions:**

Pcom-MANs may be a useful disease model to investigate possible causes of aneurysm rupture. The ruptured aneurysms manifested lower WSS, higher percentage of low WSS area, and higher AR, compared with the unruptured one. And hemodynamics is as important as morphology in discriminating aneurysm rupture status.

## Introduction

For a variety of reasons, an increasing number of asymptomatic unruptured intracranial aneurysms (IAs) have been discovered recently. Although unruptured cerebral aneurysms bear a relatively low risk of rupture, [1], [2] preventive interventions are commonly performed due to the poor prognosis of aneurysm rupture. However, given the significant potential risks of treating unruptured intracranial aneurysms (in some groups of patients, the treatment risks may outweigh the aneurysm rupture rate) on the one hand, and the known serious morbidity of aneurysm rupture on the other hand, it is a common dilemma to determine which aneurysms that demand prophylactic therapies. Therefore, it is highly desirable to predict aneurysm outcomes and select those lesions with high rupture risks for further treatment.

The mechanisms underlying aneurysm ruptures are not well understood yet. Hemodynamic factors are commonly believed to play an important role in the pathogenesis, progression, and rupture of cerebral aneurysms. With the advancement of 3-dimensional imaging and computational fluid dynamics (CFD) technology, patient-specific hemodynamic analysis has become feasible.[3]–[5] Intra-aneurysmal hemodynamic factors, such as wall shear stress (WSS), Low WSS area (LSA), impingement regions, inflow jet sizes, and oscillatory shear index (OSI), have been proposed as indicators for IA rupture risk.[3]–[6] However, hemodynamics may be affected by some specific factors, such as boundary conditions and vessel morphology. Generally, it is very difficult to obtain the patient-specific boundary conditions in clinical applications. In this context, analyzing the risk of aneurysm rupture with mirror aneurysms (MANs), defined as bilateral saccular aneurysms at roughly the same locations on each side in the same patient in accordance with G. Lu et al [7], provides an ideal internal control for such variables as blood pressure, age, collagen genetics and so forth. But the morphology of aneurysm and parent artery exerts a more significant influence on hemodynamic results than the boundary conditions. [8] Many scholars have been researching on the relationship between aneurysm morphology and rupture. Actually, the natural courses of aneurysms, induced by different locations, are considerably different. The researches on the relevance between these and hemodynamics are still needed. [9] The study on the morphology of parent arteries has also proved its close correlation with hemodynamics. [6] Therefore, taking advantage of MANs, the morphology-based hemodynamic study has a great significance to explore the hemodynamic mechanism of aneurysm ruptures. In this study, we chose the Pcom-MANs for morphological and hemodynamic research, so as to discover the dangerous factors of rupture.

## Materials and Methods

The Institution Review Board of Changhai Hospital, affiliated to the Second Military Medical University, approved this retrospective study, and the requirement for informed consent was waived. In addition, we have not conducted research outside our country of residence.

### Patients and Imaging

From July 2009 to April 2012, 10 patients with intracranial posterior communicating artery (Pcom) MANs were diagnosed by RDSA at our institution. Two patients without ruptured aneurysms were excluded. The remaining 8 patients with Pcom-MANs were included in this study and divided into 2 groups (i.e. ruptured and unruptured groups). Ruptured aneurysm was determined by the size of the aneurysm, the regularity of its shape and the site of hemorrhage shown in CT scan. Patients’ ages ranged from 51 to 74, with a mean age of 63.1 years. In these 8 patients, 7 were female and only one was male. And the ruptured sides of 6 included patients were the left.

The images of these 8 pairs of Pcom-MANs were obtained from Integris Allura Flat digital subtraction angiography (Philips Healthcare, Best, the Netherlands). Rotational angiograms were performed 1-second after a 9-second contrast injection for a total of 22.5 mL of contrast agent and a 180° rotation with imaging at 15 frames per second, for a total of 8 seconds. The corresponding 120 images were reconstructed on the Philips Allura FD20 workstation into 3D modeling. In order to make sure that the left and right models were consistently constructed, we performed rotational angiograms consistently, reconstructed the model by the means of obtaining the real vascular morphology, and kept the threshold level of the iso-surface segmentation in the same range. In addition, we compared the reconstructed model with the measurement of 2D image in the same position to reproduce the geometry of the model as real as possible.

The pulsatile velocity waveform was obtained by transcranial Doppler from a normal subject. We then captured the flow spectrum envelope to obtain average blood flow velocity waveform in a whole cardiac cycle by using Matlab 7.0 software (MathWorks, Natick, Massachusetts).

### Morphological Parameter Calculation

The 3D data of a Pcom-MAN and its reconstructed geometries of the parent vessel were analyzed to provide the various morphological parameters using measurement tools in Philips Allura FD20 workstation. To ensure accurate measurements of morphological parameters, the calculation was performed by two skilled neurosurgeons [YY, QHH].

Five morphological parameters were investigated, including aneurysm size, aspect ratio (AR), size ratio (SR), vessel angle (VA) and aneurysm inclination angle (AIA). The three parameters (SR, VA and AIA), which incorporated the geometry of the parent vessel, were calculated as previously described by Dhar et al. [10] The definition of the viewing plane was also given by Dhar et al. [10] The IA neck plane was defined to our best ability as the location where the aneurysmal sac pouched outward from the parent vessel.

### Patient-specific Modeling of Pcom-MANs

All of the acquired RDSA images were transferred to the Philips Allura FD20 workstation (Philips Healthcare) for reconstruction and produced a virtual reality modeling language (VRML) format. And then it was converted to a stereolithography (STL) format by using 3DMAX8.0 (Autodesk USA). After segmenting and surface smoothing by GEOMAGIC STUDIO 9.0 software (Geomagic USA), the surface data were imported into ICEM CFD 11.0 (ANSYS, Lebanon, New Hampshire) to create volume grids for fluid dynamics computation. Each Pcom-MAN model was meshed to create 600,000 to 1.2 million finite volume tetrahedral elements and wall prism elements (for accurate boundary layer resolution).

**Figure 1:**
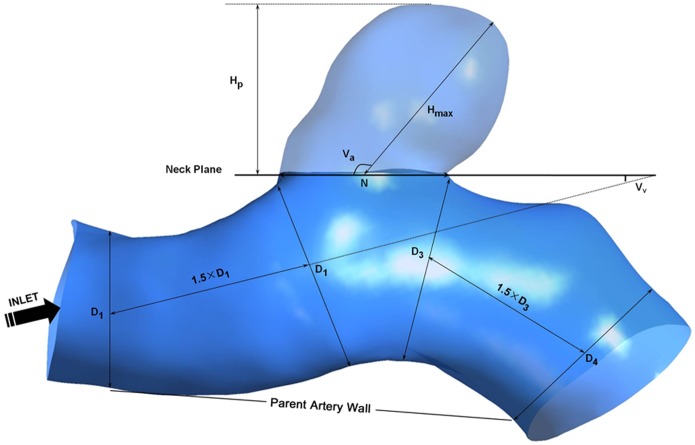
The definition of parent vessel.

The governing equations underlying the calculation were the Navier-Stokes formulations, with an assumption of a laminar and incompressible blood flow. The density and dynamic viscosity of blood were specified as ρ = 1050 kg/m^3^ and μ = 0.00345 Pa·s. We treated blood as a Newtonian fluid because non-Newtonian effects in large arteries are usually regarded as the second order. [11] The simulation was performed by CFX 11.0 (ANSYS). The vessel was assumed to be rigid with no-slip boundary conditions. The inlet was imposed by pulsatile velocity profile. All the models in our research had the same mean inflow velocity at inlet. Due to the different radius of the inlet, the inflow blood volume varied too. The radius of the inlet in our study ranged from 1.8 mm to 2.2 mm, and the mean diameter of the parent arteries ranged from 2.4 mm to 3.9 mm. The outlet was modeled as opening boundary condition with zero static pressure. We discretized the whole cardiac cycle of 0.8 second by a time-step of 0.001 second for numeric simulation. Three cardiac cycles were simulated to ensure a numeric stability, and the last cycle was taken as output. We dotted alone the neck on the 3D surface of the aneurysmal wall, and then linked them to separate aneurysms from their parent artery. The parent vessel was cut from the proximal neck with the length of 1.5 times the diameter at the proximal neck, including the upstream, downstream and branching vessels (as seen in Figure 1). And we set the pcom artery as parent artery in our research when it was involved in the aneurysm. We then postprocessed and visualized the results of these simulations by CFX 11.0 (ANSYS).

### Hemodynamic Parameter Calculation

We calculated the following hemodynamic parameters: WSS, low WSS area (LSA), OSI. And the WSS (already time-averaged, as in Eq 1.) was averaged over the dome area (the entire luminal surface of the aneurysm sac). In this study, WSS distributions were normalized by the average parent vessel WSS in the same patient to allow comparison among different patients. [12] Low WSS area (LSA), defined as the areas of the aneurysm wall exposed to a WSS below 10% of the mean parent vessel WSS, was then normalized by the dome area. [12], [13] Oscillatory Shear Index (OSI), a nondimensional parameter, measures the directional change of WSS during the cardiac cycle: [14]

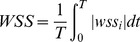
(1)

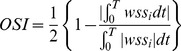
(2)where wss_i_ is the instantaneous WSS vector and T is the duration of the cycle. The OSI was averaged over the dome area.

### Statistical Analysis

The means and SDs of all morphological and hemodynamic parameters were calculated for the ruptured and unruptured groups. Data were expressed as mean ± SD. The differences between the unruptured and ruptured groups were analyzed by a paired nonparametric Wilcoxon test. A P value <0.05 was regarded as statistically significant, and all tests were 2-sided. Statistical analyses were performed using Microsoft Excel 2003, Matlab 7.0 (MathWorks, Natick, Massachusetts) and SPSS11.0 (SPSS Inc, Chicago, Illinois).

## Results

### Hemodynamic Analysis

Overall, 16 patient-specific Pcom-MAN models in the 8 included patients were constructed.

**Figure 2:**
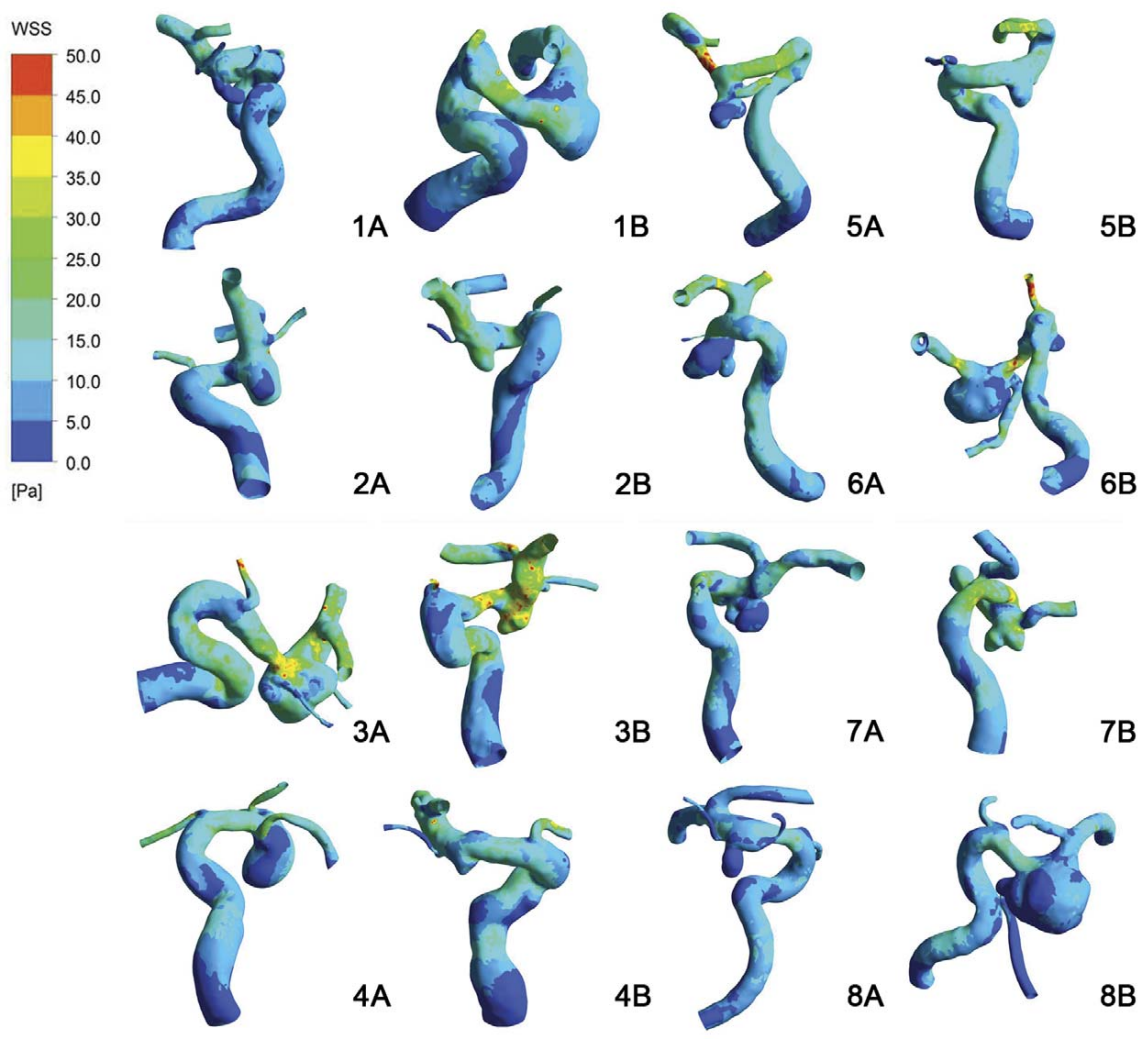
WSS distribution of 8 pairs of Pcom-MANs. A, The ruptured group. B, The unruptured group.

The distribution of WSS in the 8 pairs of Pcom-MANs was shown in Figure 2. The distribution of aneurysmal WSS was more irregular in the ruptured group. In the ruptured group, WSS values were lower within the aneurysms, especially at the domes or the daughter cysts of the aneurysms, than in the parent vessels (Figure 2), whereas in unruptured group, they were comparable. Ruptured aneurysms had lower WSS magnitudes and larger areas of low WSS than unruptured aneurysms.

The mean WSS of the aneurysm sac in ruptured group, normalized by the average parent vessel WSS in the same patient, was significantly lower than that in unruptured group (0.52±0.20 versus 0.81±0.21, P = .012).

The percentages of the low WSS area were 4.11±4.66% in ruptured group and 0.02±0.06% in unruptured group, respectively. There was a statistically significant difference between the ruptured and the unruptured groups (P = . 018).

**Figure 3:**
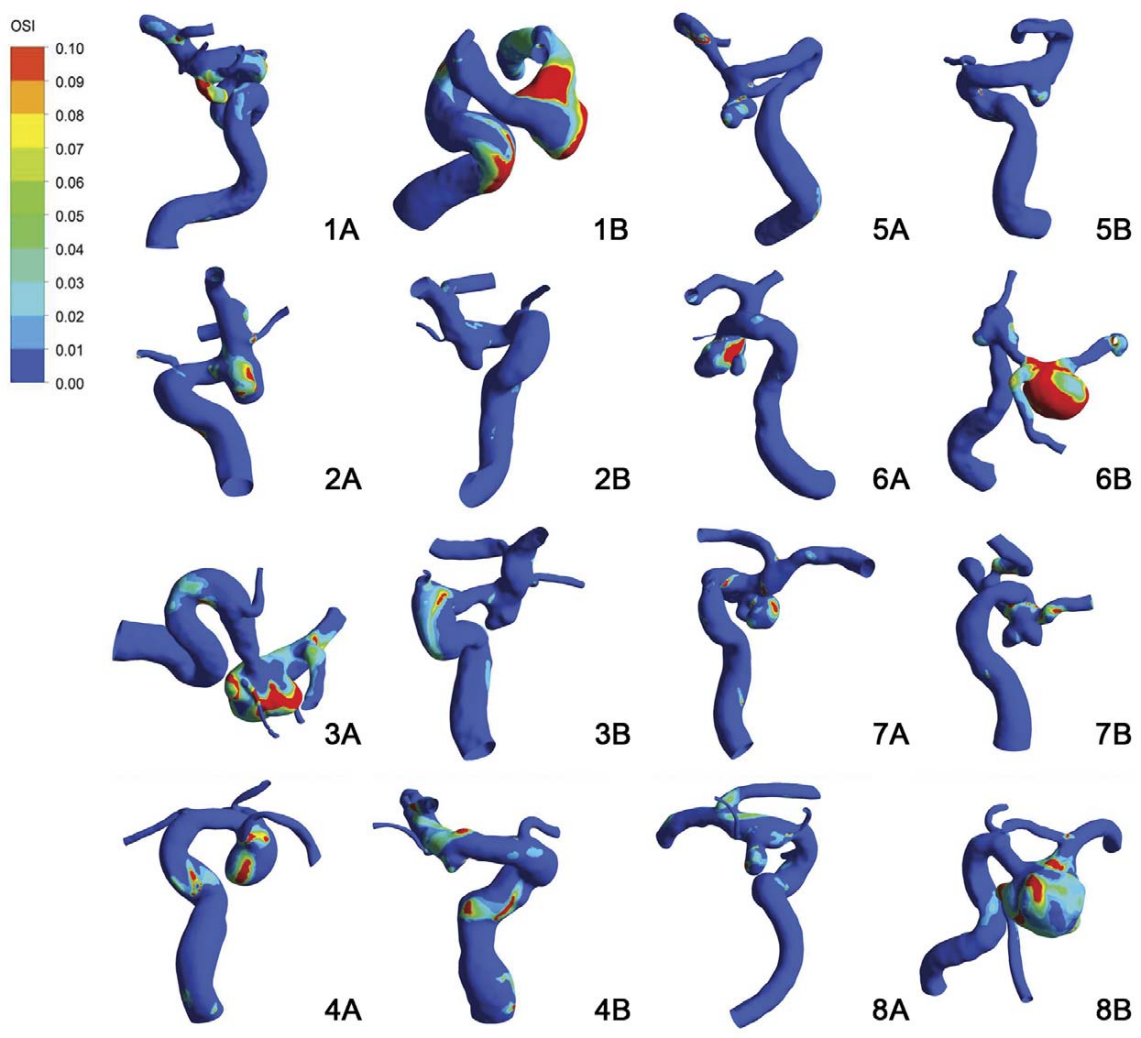
OSI distribution of 8 pairs of Pcom-MANs. A, The ruptured group. B, The unruptured group.

Figure 3 depicted the spectrum of OSI distribution of the 8 patients, with regions of increased OSI corresponding to regions of rapid WSS directional changes. However, there was no significant difference in OSI between the ruptured and the unruptured groups (0.0329±0.0318 versus 0.0456±0.0676, P = .674).

### Morphological Analysis

Values for means and SDs for each parameter were displayed in Table 1.

**Table 1:** Results From Statistical Analysis of All Parameters Examined in Ruptured and Unruptured Aneurysm Cases.

Parameter	Ruptured Mean	Unruptured Mean	P value
Size, mm	5.20±1.41	4.40±2.72	.327
AR	1.04±0.21	0.70±0.17	.012
SR	2.04±0.53	1.94±1.19	.779
VA	19.62±14.58	32.28±30.09	1.000
AIA	69.54±23.41	79.15±25.34	1.000
WSS	0.52±0.20	0.81±0.21	.012
LSA, %	4.11±4.66	0.02±0.06	.018
OSI	0.0329±0.0318	0.0456±0.0676	.674

AR stands for aspect ratio.

On average, the size of the aneurysms in ruptured group was slightly larger than that of unruptured group (5.20±1.41 mm versus 4.40±2.72 mm, P = .327). The AR of the aneurysms was 1.04±0.21 in the ruptured group, which was significantly higher than 0.70±0.17 in the unruptured group (P = .012). Although the vessel angle and aneurysm inclination angle were higher in ruptured group (VA, 19.62±14.58 versus 32.28±30.09, P = 1.000; AIA, 69.54±23.41 versus 79.15±25.34, P = 1.000), there was no significant difference. SR was 2.04±0.53 in ruptured group and 1.94±1.19 in unruptured group (P = .779), respectively.

## Discussion

Analyzing the risk of aneurysm rupture and then screening patients with high rupture risks permit possible earlier interventions. This is a sound strategy for aneurysm rupture prevention and treatment. In this study, we tested three hemodynamic parameters and five morphological parameters by using patient-specific Pcom-MANs model for correlation with IA rupture. Of these parameters, low WSS, high percentage of the Low WSS area and high AR displayed statistical significances (P<0.05).

Hemodynamic researches were most widely used in analyzing the risk of aneurysm rupture. Our results demonstrated that low WSS and high percentage of low WSS area were related to rupture. Although the relationship between aneurysm rupture and high/low WSS remains controversial in the academic community, long-term exposure to abnormal WSS predisposes the wall to weakening and rupture. Cebral JR et al. [15], [16] found that high and concentrated WSS was associated with rupture. Shojima et al. [3] reported that low WSS on the aneurysmal wall promoted growth and eventual rupture via degeneration of ECs of saccular IAs; thus low-flow conditions might induce aneurysm rupture. Studies revealing the associations between low WSS and aneurysm rupture were exemplified by the works of Goubergrits et al. [17] Xiang et al [13] proved that besides low WSS, the high percentage of low WSS area was also associated with aneurysm rupture. These findings were similar to our results. Moreover, oscillatory shear index supports the hypothesis that directional variations of shear stress on endothelial cells during the cardiac cycle could induce a pathologic response (as shown in atherogenesis). Lu et al [7] found that rupture was significantly correlated with high OSI. However, our results did not show a significant difference in this respect. This might be due to the stricter inclusion criteria (the sole location of the aneurysms ) in our study as well as the relative small sample size.

In recent years, CFD has become a powerful and desirable tool in the investigation of neurovascular diseases. Patient-specific hemodynamic computations, embodied as fluid dynamics computations by using patient-specific information for geometry, greatly enhance our ability to predict aneurysm rupture.

The hemodynamic research, based on patient-specific DSA images for geometry and patient-specific boundary conditions, is most desirable. Thanks to the technical progress of 3D imaging technique and computer, recently idealized aneurysm models have been converted to patient-specific aneurysm models in the numerical simulation research of aneurysms. Although PC-MR helps to obtain patient-specific boundary conditions, its accuracy still needs further validations. In a retrospective study of a large sample test, it is difficult to obtain the boundary conditions for each patient. Additionally, a number of factors are believed to influence the setting of boundary conditions, such as age, sex, weight, blood pressure, etc. Eliminating these effects as much as possible is the key to enhancing the precision of CFD results when the specific boundary conditions are failed to be obtained. Adopting MAN models to compare the hemodynamic factors between ruptured and unruptured groups in the same location within the same patient, we can achieve the consistency of boundary conditions as much as possible. To some extent, this can overcome the impact of case selection bias.

Compared with hemodynamic analysis, it is easier to obtain the morphological parameters from clinical cases. Morphology is used as a critical factor in judging the responsible focus in multiple aneurysm cases. Therefore, many researchers have analyzed geometry parameters of IAs in order to capture the characteristic hemodynamics and potentially predict rupture risks. Aneurysm rupture related factors, including size, AR, etc, have been extensively investigated. The relationship between IA rupture risk and IA size is controversial.[18]–[22] But aspect ratio (AR) is a kind of shape index that has been proposed in previous studies. It has been widely studied and has consistently been found to correlate with IA rupture. [22], [23] Researchers have struggled to agree upon an optimal AR threshold value above which IAs may be deemed as dangerous. The threshold value of AR obtained from the study performed by Dhar et al. [10] is 1.18, compared with 1.6 reported by Ujiie et al. [24] Our research has further confirmed the correlation between AR and aneurysm rupture, since AR in ruptured group was significantly higher than that in unruptured group. Till now, many researchers have believed that AR is one of the key indexes to discriminate the risk of aneurysm rupture. But most of the studies were based on multi-located aneurysms, which may lead to different results. Our research showed the value of AR was 1, which was different from others. This may be related to the inclusion of single located pcom aneurysm and the relative small sample size. It might be more meaningful to research on the same location with a larger sample size. The cases included in previous studies were complex, and they did not take the curvature of parent vessel into account. The relationship between parent artery and aneurysm might have a significant influence on IA rupture. SR, vessel angle and aneurysm inclination angle are important reflections of the morphology of parent artery. Dhar et al. [10] found that aneurysm angles and SR were statistically significant for rupture, while the vessel angle did not show a statistical significance. The study of Merih I et al. [25] concluded that inflow-angle was a significant discriminant of rupture status in sidewall-type IAs. However, our results showed that the above three vessel morphological parameters had no significant correlations with rupture. This might be ascribed to the small sample size, as well as the influence of Pcom artery morphology. As the IAs in our study were all located on the posterior communicating artery, their parent arteries had a similar size. Meanwhile the shape deformation or the degradation of the posterior communicating artery might be strong. It is not suitable for us to consider aneurysm maximum height in relation to the parent artery as a morphological parameter in our study. Yet, it is meaningful for us to analyze morphological parameters like AR, regarding the IA itself only.

Hemodynamics of IA can be affected by many variations. Cebral et al. [8] showed that hemodynamics with a given geometry did not vary significantly with physiological variations of flow rate, blood pressure, and waveform. It can be inferred that IA hemodynamics might be strongly dependent on the geometry of the aneurysmal sac and its parent vessel. [6], [26], [27] Ujiie et al. [27] speculated that the relevance between AR and aneurysm rupture was owing to differences in intra-aneurysmal ﬂow. Zeng et al. [28] found differences in values and distributions of hemodynamic parameters between low and high AR cases with rabbit models. Moreover, the geometric and hemodynamic relevance of the rabbit model to human IAs has been previously demonstrated. [29] Tremmel et al. [30] have demonstrated that aneurysmal flow pattern becomes more complex and the area of low WSS increases with increasing SR. This trend is consistent with the data reported by Xiang et al. [13], in which SR was inversely correlated with WSS. Therefore, even if Pcom-MANs or patient-specific boundary conditions were included in the research, it would still not be enough for us to analyze the rupture risks of aneurysms only from hemodynamic point of view. In future studies, it is important to include both morphological and hemodynamic parameters and test them in larger groups. There is also a need for studies that identify the interrelationships between morphology and hemodynamic parameters and the mechanistic pathways by which these surrogates contribute to aneurysm rupture.

### Limitations

There are some limitations in this study. Firstly, although Pcom-MAN models based on patient-specific DSA images were used, we did not use patient-specific boundary conditions, which might cause deviations of the research. Secondly, as the prevalence of MANs is relatively low, our study is a small sample test of 8 pairs of MANs. Another concern is that the IAs in our study were all located on the posterior communicating artery. The great variety of the posterior communicating artery may affect the results of our research. Furthermore, although CFD allows us to formulate mechanistic hypotheses about the process of aneurysm evolution and test them with clinical data, CFD has multiple limitations at present. Compared with intuitive morphological analysis, CFD is time-consuming and professionally challenging.

### Conclusions

Multiple morphological and hemodynamic factors may be related to rupture of IAs, including lower WSS, larger percentage of low WSS area and higher mean AR. Pcom-MANs are a useful disease model in which many factors are equaled to explore the possible causes of rupture. The relative contributions of morphology and hemodynamics, their possible correlations, and the ability of these markers to predict rupture risks need to be assessed in larger prospective studies that include follow-ups of patients with unruptured aneurysms.
